# Cognitive Reserve in Young and Old Healthy Subjects: Differences and Similarities in a Testing-the-Limits Paradigm with DSST

**DOI:** 10.1371/journal.pone.0084590

**Published:** 2014-01-03

**Authors:** Josef Zihl, Thomas Fink, Florian Pargent, Matthias Ziegler, Markus Bühner

**Affiliations:** 1 Department of Psychology, Ludwig-Maximilians-Universität, Munich, Germany; 2 Max-Planck-Institute of Psychiatry, Munich, Germany; 3 Institute of Psychology, Humboldt Universität zu Berlin, Berlin, Germany; 4 Department of Psychology, Ludwig-Maximilians-Universität, Munich, Germany; Cardiff University, United Kingdom

## Abstract

Cognitive reserve (CR) is understood as capacity to cope with challenging conditions, e.g. after brain injury or in states of brain dysfunction, or age-related cognitive decline. CR in elderly subjects has attracted much research interest, but differences between healthy older and younger subjects have not been addressed in detail hitherto. Usually, one-time standard individual assessments are used to characterise CR. Here we observe CR as individual improvement in cognitive performance (gain) in a complex testing-the-limits paradigm, the digit symbol substitution test (DSST), with 10 repeated measurements, in 140 younger (20–30 yrs) and 140 older (57–74 yrs) healthy subjects. In addition, we assessed attention, memory and executive function, and mood and personality traits as potential influence factors for CR. We found that both, younger and older subjects showed significant gains, which were significantly correlated with speed of information processing, verbal short-term memory and visual problem solving in the older group only. Gender, personality traits and mood did not significantly influence gains in either group. Surprisingly about half of the older subjects performed at the level of the younger group, suggesting that interindividual differences in CR are possibly age-independent. We propose that these findings may also be understood as indication that one-time standard individual measurements do not allow assessment of CR, and that the use of DSST in a testing-the-limits paradigm is a valuable assessment method for CR in young and elderly subjects.

## Introduction

The concept of cognitive reserve (CR) has been designed to describe and explain individual differences in susceptibility to pathological changes in brain morphology in subjects suffering from traumatic brain injury [1], and in elderly subjects with brain atrophy [2], [3]. In contrast to the traditional view, cognitive performance in these elderly subjects was substantially better than expected, indicating “some people to be more resilient to brain changes than others” [4], p. 1006. Meanwhile the concept of CR is widely accepted to explain the mismatch between expected and observed cognitive capacities also in other pathological conditions, e.g. cerebrovascular disease [5], Parkinson’s disease [6], white matter disease [7] and multiple sclerosis [8], and is interpreted in terms of “potential buffers between brain pathology and disease outcome” [9], p. 122. Various factors contribute differently to CR, e.g. linguistic ability [10], educational and occupational attainment as well as leisure activities and lifelong experiences [4], [11], lifestyle including cognitively demanding activities [12], dietary habits and regular physical exercise [13], and mentally beneficial activities [14]. Motivation-related occupational abilities [15], regular challenging cognitive activities as well as higher socioeconomic status are associated with reduced risk of mild cognitive impairment (MCI) and dementia [16]. However, as Satz et al. [9] have pointed out, no construct validation has been proposed that allows empirical testing of the role of the specific biological and non-biological indicators of CR. In their hypothesized four-factor model of CR capacity, general intelligence (“g”), complex mental activity, processing resources, and executive functioning represent the “potential reserve proxies”, each with several specific indicators. In combination with the hypothesised brain reserve capacity model, this conceptual framework is undoubtedly an important step forward because it allows empirical testing of the significance and role of the different components and indicators. However, CR is typically viewed as a more or less ‘mechanistic’ capacity, i.e. all indicators proposed so far represent either rather static variables or reflect compensatory means for brain pathology, both with undefined degree of modifiability and thus adaptability. The use of brain morphology variables and/or (mental) proxy measures, for example, education, occupation, leisure activities, etc. as indicators of CR may be too “passive” in nature [3]. If one accepts Stern’s view [3], that CR allows subjects to cope with functional consequences of unfavourable functional brain alterations “by using pre-existing cognitive processes or by enlisting compensatory processes” (p.2016), then one would prefer a more dynamic definition of CR. Jones et al. [17] pointed out, that CR “may be a potentially modifiable characteristic, for example through mental or physical exercise” (p. 599). The participation in mentally stimulating activities is a highly robust correlate of CR [18], indicating that regular practice of mental functioning is supportive for maintaining cognitive performance [19]. However, cognitive resources representing CR may not be fully activated in routine task conditions, but are activated when required for flexible and successful response to non-routine task conditions, which imply mental challenges. The question then arises whether a more dynamic type of assessment would be helpful to determine CR in a more direct way by measuring the individual, differential activation of CR when the subject is confronted with a cognitively demanding challenge, and may thus be expected to “boost” CR [17]. A particularly helpful methodological approach to assess CR as understood here is the testing-the limits paradigm, i.e. the measurement of potential boosting of performance after practice with a cognitive task, because the “standard one-time-assessments may not reflect the latent competence in the range of plasticity” [20], p. 351. This experimental approach has been found useful in proofing the presence of latent cognitive capacities in elderly subjects [21], [22], [23].

In the current study we were interested in proving the usefulness of a complex cognitive task, the digit-symbol-coding test (also known as digit symbol substitution test, DSST) for the assessment of CR in a group of 140 younger (mean age: 23 yrs) and 140 older healthy adults (mean age: 67 yrs). Performance in the DSST is relatively unaffected by intelligence (g), memory, or learning capacity ]24, pp. 368], but performance in this test was found to be sufficiently sensitive for mental ageing independent of years of education [25]. Expected individual improvements in performance were assumed to indicate activation of individual resources for the given complex cognitive challenge. Furthermore, we assessed broader individual cognitive ability baseline, mood and personality traits. The following main questions were addressed: (1) Is the DSST a useful and robust testing-the-limits paradigm for assessing CR in younger and older healthy subjects? (2) What are the essential differences in the degree and temporal course of CR in younger and older subjects, and in variability of practice effects between and within the two groups? (3) Do one-time standard individual assessments predict CR, i.e. do high-performing subjects at baseline also show higher CR? (4) Which cognitive (cognitive speed, working memory, cognitive flexibility and visual problem solving) and non-cognitive factors (mood, personality traits) influence CR? Research concerning the development of fluid and crystallized intelligence in general has focused on Openness to experience. The OFCI model proposed by Ziegler et al. [26] shows that Openness has a direct positive effect on fluid intelligence through environmental enrichment. Moreover, an indirect effect for Openness on crystallized intelligence could also be confirmed. Thus, personality traits such as Openness have been shown to play a role in maintaining or improving cognitive ability. Therefore, personality as a predictor of CR will also be focused on in this study. For better differentiation and understanding, we use the term ‘cognitive architecture’ to denote cognitive performance in standard one-time assessments, without implying that the cognitive functions in question belong to either fluid or crystallised intelligence. In fact, our understanding of cognitive architecture is based on the idea that cognitive performance per se relies necessarily on the existence of cognitive functions and their development during life as guided by experience, and is geared to the model of Anderson et al. [27] with several modules, and their interactions, involved in and subserving cognitive functioning. In contrast, CR is defined here as the dynamic improvement in performance after systematic practice assessed by repeated measurements.

## Materials and Methods

### Participants

This study was approved by the ethical committee of the Medical Faculty at Ludwig-Maximilians-Universität, Munich. Written consent was additionally obtained from all subjects. In total, 298 younger and older healthy adults participated voluntarily in this study. All participants had at least 13 years of education. Younger subjects (n = 140; 100 female, 40 male, age: 20 to 30 yrs, *M* = 22.81 yrs, *SD* = 2.41) were recruited mostly via flyers among students from two large southern German universities. Ten younger subjects were excluded because they were either already familiar with the test material used (*n* = 5) or because of difficulties with compliance (*n* = 5). Older subjects (n = 140; 66 females, 74 males; age: 57–74 yrs, M = 67.27, *SD* = 4.16) were recruited from a larger sample (n = 148) of Senior University students. Eight older subjects were not included in the study/data analysis, either because of meeting exclusion criteria (health problems; n = 3), or because of difficulties with compliance during testing (n = 5). 121 of the older participants had a University degree (18 years of education) or doctoral degree (21 years of education).

Before entering in the study, a detailed telephone interview was conducted with potential participants for screening of exclusion criteria, in particular health problems (cardiac, metabolic, or endocrine insufficiencies, neurologic or psychiatric disease) and medication as well as non-correctable visual or auditory impairments which could interfere with cognitive performance. Participants were reminded to abstain from alcohol and medication that might interfere with cognitive processes, as well as to abstain from drinking coffee on the day of participation no later than one day prior to the scheduled testing session.

### Assessment of Cognitive Architecture

Cognitive architecture was assessed on the basis of standard-one-time measurements of information processing/attention (digit cancellation test d2; [28]), verbal short-term and working memory (subtests for digit spans forward and backward; [29]), and visual problem solving (Matrices of the WAIS-III; [30]).

### Measurement of CR

Systematic practice effects were determined using the Digit Symbol Substitution Test (DSST; [30]). The DSST is a paper and pencil test. Participants are presented with nine symbols, each representing one of nine (1–9) digits. A series of digits with a blank space for sketching the symbol underneath is presented on the same sheet of paper. Subjects are asked to assign as many symbols as possible to the respective digits. The test was administered ten times consecutively with exactly the same order of symbols. To avoid ceiling effects, the time for each repetition was reduced to 90 seconds (standard: 120 seconds). Between repetitions, participants were given a break of 1 min to prevent fatigue effects of the hand. The number of correctly assigned and written symbols was used as performance measure.

### Other Measures

In addition to the cognitive tests, a socio-demographic interview was carried out, which also included questions concerning physical and mental health. Mood was assessed in the group of younger subjects with the Montgomery-Asberg Depression Rating Scale (MADRS; [31]), whereas for the older participants Beck’s Depression Inventory – Second Edition (BDI-II; [32]) was used. For the assessment of personality traits, the computerized German version of the NEO-Five-Factor-Inventory (NEO-FFI; [33]) was used in both groups.

The assessment of cognitive and non-cognitive variables and the CR lasted approximately two hours. One psychologist and four well trained and regularly supervised student assistants performed the assessment. For the participation in the study participants received 30 Euro as financial compensation after the assessment was completed.

### Data Analysis

#### Calculation of indexes of cognitive reserve

There is no unequivocal definition of gain scores after practice trials [34], [35], [36], [37]. Williams and Zimmermann [34] have argued that simple gain scores can be very useful in research. Thus we decided to use two different measures of gain after systematic practice in ten consecutive trials in the DSST as terms for CR: the raw gain score defined as the difference between best performance (highest number of correct items) and the performance in the first trial, whereby the best trial was not necessarily the last trial. The second measure for CR was calculated using the following formula:
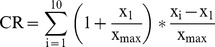


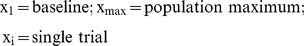



This measure can be understood as the area under the curve of the relativized gain function. The raw gain score is first divided by the maximum gain score of the respective population and then multiplied by the quotient of the baseline and maximum gain score. This calculation method has several advantages. Ceiling effects in improvement are relativized by compensating for the baseline, comparability of CR measures is improved by including the maximum gain score of the population, and complete progression of the improvement function is taken into account by utilizing the integral of the function. The output of the formula is always 0 for the first trial, as it represents the baseline, then increases (positive values) or decreases (negative values) with relative increment or decrement in DSST performance. In the following, this measure will be referred to as modified gain score.

#### Statistical analysis of data

All data were analysed using IBM® SPSS® Statistics 20. Differences in baseline, raw gain, modified gain scores and consistency measures were calculated with independent-samples t-tests. To analyse differences between trial numbers required to reach defined performance levels a repeated-measures ANOVA (analysis of variance) was carried out. Bivariate Pearson-product moment correlations were performed to test relationships between modified gain scores and cognitive architecture and personality traits, respectively. All reported p-values are Bonferroni corrected.

## Results

### Cognitive Architecture

Table 1 shows age and gender, and outcome of cognitive architecture assessment. The younger group of participants scored significantly higher on the digit cancellation test (t[271.47]) = 13.35, p<.0005, d = 1.59), digit spans forward (t [278]) = 6.32, p<.0005, d = .75) and backward (t[268.94] = 6.11, p<.0005, d = .73) and Matrices test (t[222.70]) = 11.34, p<.0005, d = 1.35) than the older group.

**Table 1:** Demographic description of the two subject groups and outcomes in cognitive architecture.

	Younger adults	Older adults
**Age**	22.81 (±2.41)	67.27 (±4.16)
**Gender**	40 m, 100f	74 m, 66f
**d2**	201.68 (±37.46)	146.07 (±32.04)
**DS forwards**	8.77 (±1.77)	7.49 (±1.61)
**DS backwards**	8.94 (±2.05)	6.56 (±1.71)
**SPM**	21.81 (±2.58)	16.88 (±4.45)

Mean test scores and ±1 standard deviation in brackets, age in years, m = males, f = females, d2 = digit cancellation test, DS = digit spans, SPM = Standard Progressive Matrices.

### Cognitive Reserve

**Figure 1:**
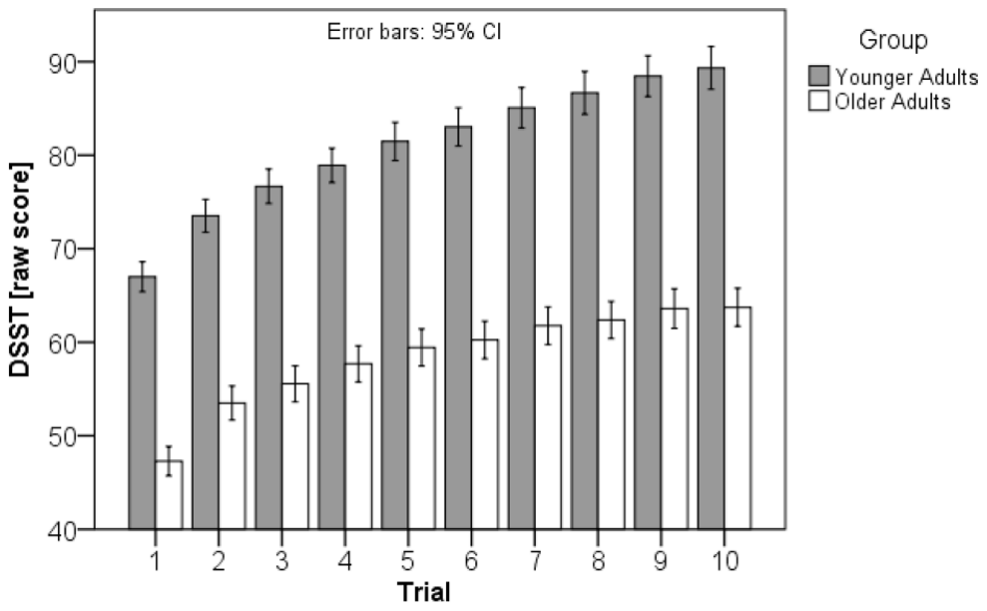
DSST raw scores of the younger and the older group in 10 consecutive trials in the DSST. Note the difference in base line performance between groups, but the similar increase in performance in both groups. Vertical bars indicate 95% confidence intervals.

**Figure 2:**
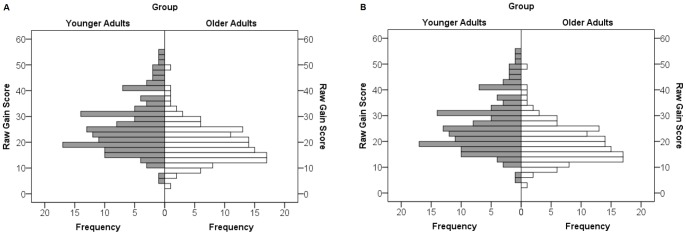
Histogram of the raw (left) and modified gain scores (right) of the younger and older group. Note the large overlap in gain scores for younger and older subjects in both gain measures.

Figure 1 shows the outcome of systematic practice with the DSST over 10 trials. In both groups the increase in performance was substantial with large within variations of practice effects (see Figure 2). In the first trial, the younger group of subjects processed correctly on average 67.01 (SD = 9.63) items; on average the best performance in this group was 89.34 (SD = 13.67) correct items. The older group of subjects processed on average 47.28 (SD = 9.36) items correctly in the first trial; their best average performance was 63.74 (SD = 12.22) correct items. Thus, the raw gain score in the younger group was 25.52 (SD = 10.10) and 18.94 (SD = 7.30) in the older group. The modified gain score was 1.72 (SD = .81) for the younger group, and 1.24 (SD = .59) for the older group, respectively. Baseline scores and gain scores differed significantly between groups (baseline: t [278] = 17.38, p<.0005, d = 2.07; gain raw scores: t [278] = 6.26, p<.0005, d = .75; modified gain scores: t [278] = 5.60, p<.0005, d = .67). Thus the younger group shows significantly higher baseline scores as well as significantly higher gains. The two measures of gain, the raw and the modified gain scores, are highly significantly correlated with each other (r = .95, p<001).

For a more detailed characterization of practice effects in the two groups the number of trials was calculated that was required to achieve a particular intraindividual level of performance. The younger group reached the 50 percent level of increase in DSST performance on average after 4.46 trials (SD = 1.56), the older group after 3.77 (SD = 1.35) trials. For statistical analysis, a repeated-measures ANOVA was carried out with ‘group’ as first factor and ‘mean trials’ required for achievement of 50, 75 and 90 percent of the respective performance level, as indicated by gain raw scores, as the second factor. We found significant main effects for group (F [1, 139] = 15.54, p<.0005, ) and number of trials (F [2, 278] = 668.45, p<.0005), but no significant interaction between the two factors (F [2, 278] = 1.55, p = .22). Post hoc tests using the Bonferroni correction revealed that the differences between the 50 and 75 percent (p<.0005) as well as between the 75 and 90 percent performance levels (p<.0005) were significant. t-tests with Holm-Bonferroni correction showed significant differences between groups for the 50 percent (t[272.23] = 3.93, p<.002, d = 47), 75 percent (t [278] = 3.46, p<.002, d = .41) and 90 percent performance levels (t[289.58] = 2.18, p<.03, d = .26). Thus, interestingly, the younger group required significantly more trials to achieve all performance levels.

### Similarities between Groups

Apart from differences between groups, we were also interested in similarities in performance characteristics, which became evident by analyzing performance of younger adults with average performance and identifying their counterparts in the ‘older’ group. For this purpose subjects from both age groups with a modified gain score within the second and third quartiles of all values (equal to all values from 1.09 to 2.21 in modified gain score; see Figure 2) were selected. On this basis, 70 younger and 72 older adults were included into the statistical analysis. There was only a small, but not significant difference in modified gain score between these two subgroups (t [140] = 1.65, p = .10, d = .20). In other words, 72 older subjects (51.4%) performed at the same level as 70 (50%) younger subjects, but 48.60% of the older adults performed at a lower level. It should be added, that neither in the younger (r = -.12, p = .17) nor in the elder group (r = .09, p = .29) there is a significant correlation between the baseline and the raw gain scores.

### Consistency of Gains

#### Intraindividual variability

**Figure 3:**
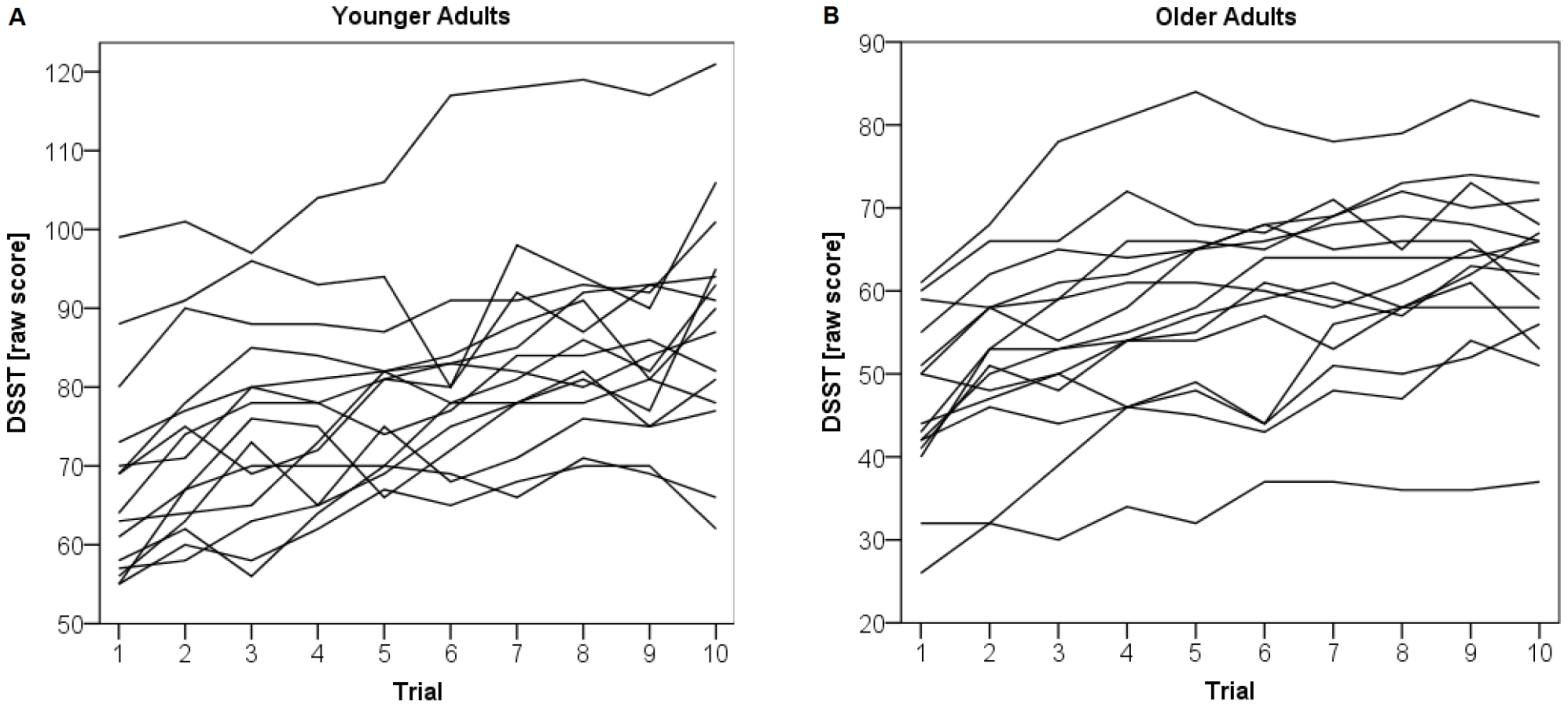
Exemplary performance curves of 10 consecutive trials in the DSST of 15 randomly selected younger subjects (left) and 15 randomly selected older subjects (right). Note differences in baseline and in interindividual performance variation in both groups.



Figure 3 shows examples for intraindividual variability in performance in the DSST in 15 younger and 15 older randomly selected subjects. Interestingly, some subjects show small variations in performance, while others show rather high variability. One way of analyzing the consistency of gains in the DSST in the two groups is to calculate and compare performance deteriorations. For this purpose the frequency of trials with lower correctly processed items compared with the preceding trial was counted. In the younger group 2.43 (SD = 1.11) of such trials were found on average; the corresponding rate in the older group was 2.49 (SD = 1.12) trials. The difference between the groups is not significant (t [278] = .42, p = .68, d = .05). West et al. [37] have proposed an alternative possibility for investigating the inconsistency of performance: deviations from individual performances are relativized by the performance of the current trial to compensate for different levels of performance, according to the formula:

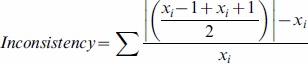


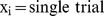



**Figure 4:**
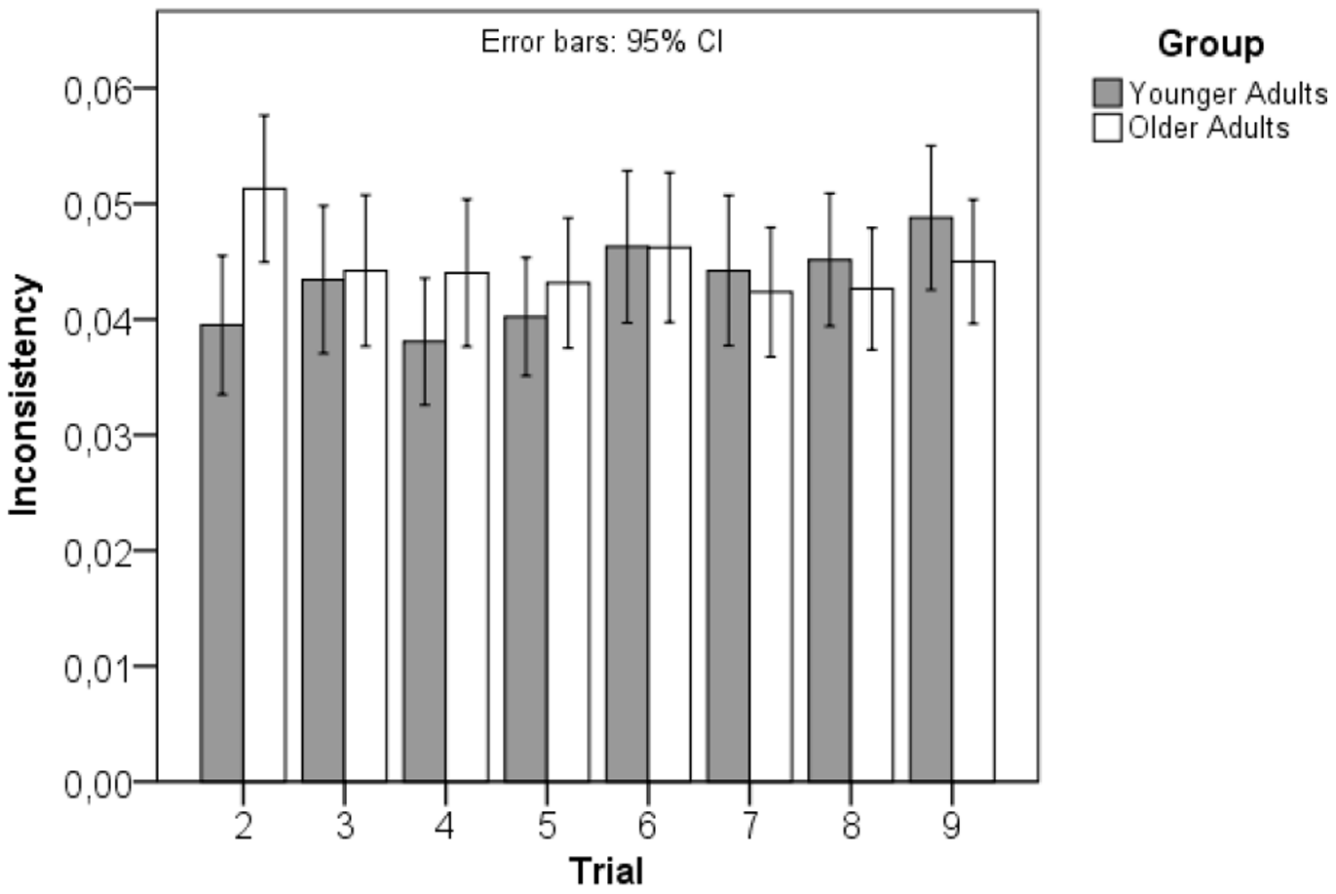
Inconsistency values for DSST raw gain scores for the younger and the older group in the 2nd to 9th trial. Note the similar inconsistency in all trials for both groups. Vertical bars indicate 95% confidence intervals.

On the basis of this formula the younger group showed a mean inconsistency value of.34 (SD = .15), the older group of.36 (SD = .15); both measures do not differ significantly (t [278] = .68, p = .50, d = .08; see Fig. 4).

#### Interindividual variability

**Figure 5:**
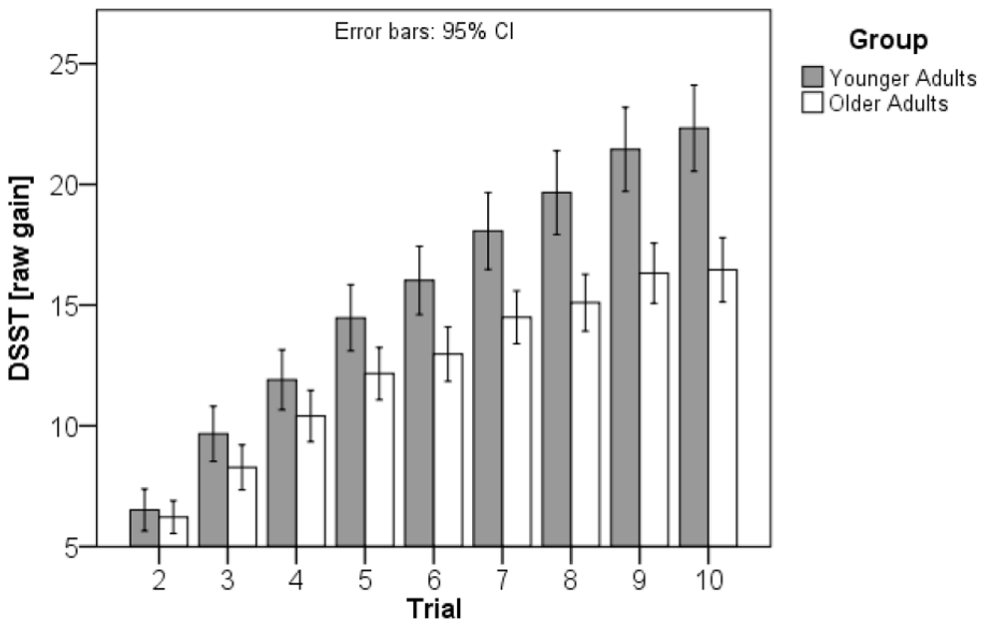
Interindividual variability in DSST raw gain scores for the younger and the older group in the 2nd to 10th trial. Note the similar gain in the first 4 trials and increasing differences in the later trials. Vertical bars indicate 95% confidence intervals.

Interindividual consistency in performance was tested by means of Levene’s tests. Variability in the DSST baseline performance did not differ significantly between groups (F [278] = .02, p = .90). However, the variance of the raw gain scores (F [278] = 13.96, p<.0005) and of the modified gain scores (F [278] = 16.06, p = .0005) differed significantly between the two groups, with the younger subjects exhibiting significantly larger gain differences with increasing number of trials (see Figure 5).

### Relationship between Cognitive Architecture and Modified Gain Score

Correlations between the modified gain score and measures for information processing/attention (d2), verbal short term (digit span forward) and working memory (digit span backward), and visual problem solving (SPM) were calculated. As can be seen in Table 2, in the younger group no correlation was significant (highest p = .14) and effect sizes were rather small (highest r = .15). In the older group, information processing (r [138] = .35, p<.001), verbal short term memory (r [138] = .20, p = .04) and visual problem solving (r [138] = .19, p = .05) were significantly correlated with the modified gain score. The correlations with verbal short term memory and visual problem solving possess small to medium effect sizes, and the correlation with information processing a medium to large effect size.

**Table 2:** Correlations between modified gain scores and measures of cognitive architecture.

	Younger Group		Older Group	
	r	p	r	p
**d2**	.09	.60	.35***	.00
**DS forwards**	.15	.14	.20*	.04
**DS backwards**	.07	.87	.16	.13
**SPM**	.15	.17	.19*	.05

All p-values are Bonferroni corrected, * signals a significant correlation on the.05 level (1-tailed) *** signals a significant correlation on the.001 level (1-tailed), r = correlation coefficient, p = p-values, d2 = digit cancellation test, DS = digit spans, SPM = Standard Progressive Matrices.

### Relationship between Personality Traits and Modified Gain Score

To examine the relationship between CR and the Big Five personality traits (Neuroticism, Extraversion, Openness to experience, Conscientiousness and Agreeableness) correlations with power values were calculated (see Table 3). These correlations reached neither in the younger nor in the older group significant levels and showed relatively low effect sizes.

**Table 3:** Correlations between modified gain scores and personality traits.

	Younger Group		Older Group	
	r	p	r	p
**Neuroticism**	.10	.99	.14	.53
**Extraversion**	−.18	.16	−.11	.90
**Openness**	.10	.99	−.01	.99
**Agreeableness**	−.16	.99	−.17	.25
**Conscientiousness**	.07	.99	−.08	.99

All p-values are Bonferroni corrected, r = correlation coefficient, p = p-value, note that no correlation is significant.

### Relationship between Cognitive Architecture, Modified Gain Score and Mood

We did not find significant correlations between mood scores and measures of cognitive architecture or modified gain scores in the DSST, neither for the Montgomery-Asberg Depression Rating Scale in the younger group nor for the Beck’s Depression Inventory in the older group (highest p = .35, highest r = −.125).

## Discussion

The main outcome of this study is that both, younger and older subjects showed a significant increase in performance in the DSST after systematic practice. Baseline performance and increase after practice were, however, significantly higher in the younger group. Furthermore, cognitive performance in traditional testing conditions (i.e. one-time assessment), i.e. cognitive architecture, was also significantly higher in the younger group for information processing speed/attention, verbal short-term and working memory and visual problem solving. These results are in line with reports suggesting the persistence of individual differences in cognitive functioning rather than differential rates of age-associated cognitive declines [38]. Interestingly, however, the younger group required significantly more practice trials to achieve 50, 75 and 90% performance relative to the individual baseline. This resulted from the shallower learning curve of the younger group. In the older group, gain was significantly correlated with speed of information processing, verbal short-term memory and visual problem solving; in contrast, no significant correlations between measures of cognitive architecture and CR gains were found in the younger group. Gender, personality traits and mood did not significantly influence performance gains after practice, which is in contrast to other reports [39], [40], [41]. This difference in outcome may be explained by the homogeneity of our groups concerning these variables, but may also only become overt in a longitudinal study [42]. Apart from these significant differences between younger and older subjects, some interesting similarities were also found. About 50% of older subjects showed the same gain in performance as younger subjects did. In addition, consistency of increase in performance in consecutive practice trials did not differ significantly between the two age groups, indicating similar variability in both groups. However, the younger group showed a significantly higher variability in DSST performance compared with older subjects after the first few trials. This may be explained in terms of higher diversity in performance or in higher instability of performance in younger subjects. However, for a more valid assessment of intraindividual variability, longitudinal measures appear appropriate, which also consider emotional diversity and variability of biological parameters, e.g. cardiovascular and metabolic factors [42], [43]. Apart from these facts, empirical evidence from other studies supports our observations of smaller variability in cognitive performance of older adults, which has been interpreted in the context of age-associated entropy or entropy states of the brain [44], [45].

Our observations are consistent with earlier reports of significant effects of age on DSST performance (e.g. [25]) and on practice effects in healthy older subjects in cognitive tasks (e.g. [21], [23]). Furthermore, the results of this study are also in line with earlier studies reporting significant differences in performance increments between younger and older subjects [46], [47]. However, in contrast to the findings reported by Bherer et al. [48], we found significant correlations of cognitive architecture measures and improvement rates in DSST at least for the older group. Because mean age and educational level of subjects in their study was similar to our subjects, this difference in the outcome may be due to the small number of subjects (n = 12 in each group) in their study. Furthermore, it cannot be ruled out that the complexity of the task plays a significant role. Bherer et al. [48] used a dual-task paradigm to assess practice effects, which consisted of auditory frequency discrimination and a visual (letter) identification task. In contrast, the DSST task comprises many different cognitive components: visual discrimination/identification, information processing speed, visual (and probably also verbal) working memory, and executive functions (flexibility, monitoring), which have to interplay effectively to guarantee high performance and performance increase, respectively, during practice. Thus, the DSST may represent a rather complex cognitive multi-tasking condition. Interestingly, the DSST is sensitive to even minimal brain injury [49] regardless of the locus of injury [50] and is also sensitive to dementia [51] and to risk of severe hypoglycemia in type 2 diabetes [52], suggesting that impaired performance in this test is indicative of global brain dysfunction, although the frontal lobe may play an important role in this type of cognitive multi-tasking [53]. Thus, the DSST is a useful mean to measure a complex, multi-component mental operation, which may reflect CR in a sufficiently appropriate and valid form. Activation of resources underlying this complex cognitive operation by standardised systematic practice in a challenging task, as translated by the DSST testing-the-limits paradigm, may thus represent a highly potential CR proxy as proposed by Satz et al. [9]. This does not imply, that neurobiological factors subsumed under the umbrella term of ‘brain reserve’ and mental capacities per se [2] do not represent proxies of CR, but they lack the essential attribute of dynamics of CR, and thus cannot predict the outcome of challenging CR. We would like to propose, therefore, to define CR as the extent of improvement in cognitive performance in a challenging task of the testing-the-limits type, after a sufficient number of practice trials. The DSST appears a particularly suitable instrument for the standardised assessment of CR.

In conclusion, older and highly educated healthy subjects do not only possess a good level of cognitive architecture, but also retain CR, which can be used for coping with challenging cognitive tasks. This indicates that functional brain plasticity remains preserved even in older age, provided that brain reserve is sufficiently available. Cognitive architecture and CR appear, at least in our sample, largely independent of mood and personality traits in healthy younger and older individuals, but may play a significant role in pathological conditions, e.g. depression [54] or brain diseases, for example, Parkinson’ disease [6], white matter disease [7], or multiple sclerosis [8]. The difference in cognitive architecture and in CR in older as compared to younger healthy subjects is not surprising and is in line with many studies on mental ageing. However, what is surprising is that about half of our older subjects performed at the level of the younger group, and vice versa, i.e. a subgroup of younger subjects did not outperform the ‘high’ performers in the older group. This poses the interesting question of age-independent interindividual differences in cognitive architecture and probably also CR, suggesting that both are possibly not so much a question of years of life but rather of age-independent interindividual differences [55], [56].

Biological (e.g. genetic diversity, structural and functional brain efficiency) and non-biological factors (e.g. individual experiences, environmental factors) contribute to cognitive differences in humans irrespective of age [57]. In addition, there exist healthy older adults showing only slow cognitive decline over many years [58], indicating that cognitive architecture and its regular use in terms of activation of CR demonstrate high stability. Of course, as pointed out by Stern [2], [3] and Satz et al. [9], a number of factors may influence cognition and CR in a more favourable or unfavourable fashion. Apart from mental components, i.e. the absence of dementia and chronic medical states affecting CNS function, life-long use of mental capacities [16], [18], [59] and good mood [41], [42] have been identified as key factors for prolonged mental health in older ageing. A quantitative, dynamic measurement of CR may help to proof mental health span in normal and pathological conditions of the brain, irrespective of age. The use of the DSST in a testing-the-limit paradigm seems a promising approach to estimate individual cognitive resources in the context of cognitive architecture, mood, and biological variables, e.g. hormones and morphological and functional brain factors. Our results also pose some caution on the interpretation of short-term practice effects in both, younger and older subjects: the obtained improvement in cognitive performance may be due to the activation of CR rather than the result of a primary practice effect in terms of learning. Nevertheless, our results are in support of the notion that systematic repeated practice with challenging cognitive tasks can improve cognitive performance irrespective of age (e.g., [19]).

There are some limitations in our study which deserve further research and clarification. Because all our subjects had at least 13 years of education and thus all belong to the category of high education level, our data do not allow a conclusion of cognitive architecture and CR, and possible interactions, in individuals with low(er) education levels. Furthermore, it remains unclear, whether CR represents a more general (‘g’) capacity, or is also functionally specialized, which is suggested by the involvement of different components of a central circuit for complex cognition as proposed by Anderson et al. [27]. Additionally, our data do not allow commenting on the upper limit of CR, i.e. subjects may still have improved after ten trials. Finally, it would be of interest to know the dynamics of the time course of CR, for example, how long CR remains preserved once it has been activated, or whether it is activated faster when assessed a day or a week after the first activation.
